# Recurrent Solid Pseudopapillary Neoplasm of Pancreas: Case Report and Review of Literature

**DOI:** 10.1089/pancan.2018.0006

**Published:** 2018-06-01

**Authors:** Piyush K. Sharma, Sanjana Mehrotra, Ana L. Gleisner, Richard D. Schulick, Martin D. McCarter

**Affiliations:** ^1^ Department of Surgery, University of Colorado School of Medicine, Aurora, Colorado.; ^2^ Department of Pathology, University of Colorado School of Medicine, Aurora, Colorado.

**Keywords:** solid pseudopapillary neoplasm of pancreas, surgical pathology, recurrence

## Abstract

**Background:** Solid pseudopapillary neoplasm of pancreas is a rare tumor with a low potential for metastasis and recurrence. Long-term outcomes after surgical resection are excellent and recurrences after an R0 resection are extremely rare.

**Case Presentation:** We present an unusual case of a 42-year-old man who had a recurrence of his solid pseudopapillary tumor 4 years after undergoing a distal pancreatectomy and splenectomy and then again a year after his reresection.

**Conclusions:** The lack of histological features deemed to be suggestive of a malignant variant and the aggressive clinical course seen in this case is remarkable. It underscores the fact that despite the low incidence, recurrences of solid pseudopapillary neoplasms of the pancreas do occur and it can be very difficult to predict malignant potential based on radiological or histopathological features.

## Introduction

Solid pseudopapillary neoplasms of the pancreas (SPNP) are relatively rare tumors comprising around 0.13–2.7% of all pancreatic tumors and 10–15% of all pancreatic cystic neoplasms.^1^ These typically present in the second–third decade of life with a 10:1 female predominance. SPNP have a low malignant potential but local invasion can be present and metastatic spread has been reported with liver and peritoneum being the most common sites. Surgical resection affords long-term cure and the reported recurrence rate after resection ranges from 3% to 9%.^2^ We hereby present a case of SPNP recurrence in the resection bed with involvement of accessory splenic tissue, diaphragm, stomach, and omentum. The late presentation, male gender, and lack of histological features suggestive of a malignant variant and aggressive growth pattern of the recurrent tumor highlight the unique aspects of this case.

## Case Report

**FIG. 1.**
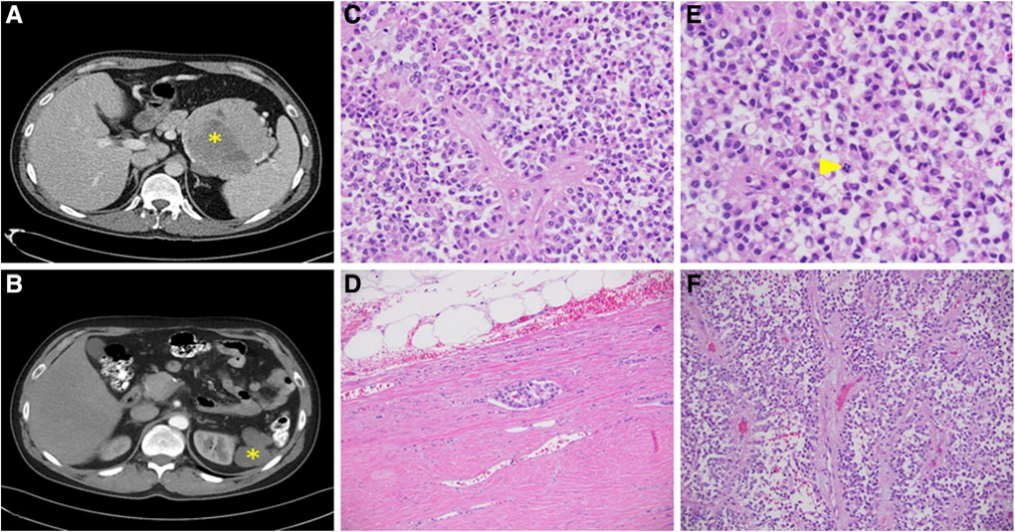
**(A)** Solid pseudopapillary neoplasm in pancreatic tail at the time of presentation, **(B)** recurrent mass in the splenic fossa. Histological features of the recurrent tumor: **(C)** characteristic pseudopapillary architecture observed on microscopy (H&E 100 × ), **(D)** lymphovascular invasion by tumor, **(E)** mitotic figures (6 in 10 high-power fields), **(F)** fibrovascular core with discohesive tumor cells.

A 42-year-old man presented to our pancreas multidisciplinary clinic after a computed tomography (CT) scan (Fig. 1A), prompted by a 2-month history of generalized bloating and epigastric discomfort, that demonstrated a 11.2 × 9.6 cm heterogeneous solid appearing mass in the tail of the pancreas. The irregular mass had several small peripheral calcifications and lobulated contours abutting the spleen, stomach, and splenic flexure of colon without any direct invasion. He underwent a distal pancreatectomy and splenectomy with splenic artery lymph node dissection. Intraoperatively the large soft lobular cystic mass at the pancreatic tail was locally contained without any obvious invasion of surrounding structures or gross metastasis. Histopathological assessment of the mass established it as a pT3pN0pMx SPNP (CD56^pos^ nuclear β-catenin^pos^ chromogranin^neg^ and synaptophysin^neg^). Margins were negative without any lymphovascular or perineural invasion. The patient was discharged home after an uneventful period of convalescence in the hospital.

Four years later, he was referred back to our clinic after discovery of a biopsy-proven recurrence in the splenic fossa (Fig. 1B). The bulk of the tumor was densely adherent to the splenic flexure and gastric fundus and was resected with wedge gastrectomy and partial colectomy. A 4 cm nodule of tumor adherent to the diaphragm as well as omentum was removed by dividing the omentum and stripping the superficial layer of diaphragm. The tumor was soft, extremely friable, and fractured with minimal manipulation. It remained densely adherent to the left diaphragm, left kidney, and left adrenal gland. Eventually, we were able to dissect down through the Gerota's fat and strip the anterior capsule of the kidney clean to dissect the tumor off the kidney and the adrenal gland. The other end of the mass remained adherent to the diaphragm and was removed along with a portion of the diaphragm.

Final pathology report confirmed the presence of recurrent metastatic SPNP in omentum, diaphragm, accessory spleen tissue, and the gastric fundus. The patient recovered well from his surgery and was discharged home. He underwent CT surveillance at 3-month intervals per his medical oncologist and his first three scans showed stable postoperative changes without any evidence of local recurrence or metastatic spread. However, his next scan showed enlarged retroperitoneal paraaortic nodes that were found to be fluorodeoxyglucose (FDG) avid. He was started on capecitabine with stable disease on recent repeat imaging in April 2018.

## Discussion

CT appearance of SPNP ranges from solid to predominantly cystic with the majority seen as a large encapsulated mass composed of a mixture of cystic, solid, and hemorrhagical components. Presence of a capsule and hemorrhagical foci within the mass help distinguish it from the other pancreatic neoplasms.^3^ Intra- or peritumoral calcifications and intravenous contrast enhancement within the mass may sometimes be present. This corresponds well with the CT findings in the case described. Histological appearance of SPNP is marked by the presence of solid, pseudopapillary, or hemorrhagical pseudocysts in varying proportions^4^ (Fig. 1C). Solid areas show sheets and cords of cells arranged around fibrovascular septa. Owing to swelling and degenerative changes, there is formation of mitotic spaces between cells farthest from blood vessels, which gives rise to the characteristic solid pseudopapillary pattern.^5^

The exact pathogenesis of SPNP remains elusive. The existing evidence suggests a role of deregulation of β-catenin pathway resulting in Sox9 and PDX1 overexpression with an associated point mutation in exon 3 of CTNNB1 gene.^6^ Such mutations are observed in 80–90% of the cases with almost all of these exhibiting the characteristic nuclear localization of β-catenin.^7^ Most SPNP cells stain diffusely positive for vimentin, β-catenin, and neuron-specific enolase, with focal staining for cytokeratin, α1-antitrypsin, α1-antichymotrypsin, and synaptophysin. Focal staining for hormonal markers such as insulin, glucagon, and somatostatin or Leu-7, LeuM1, Ki-M1P, CD34, α-inhibin, calretinin, and cholecystokinin is often seen as well, which suggests a capacity for focal neuroendocrine differentiation. Notohara et al. have reported on the potential use of CD10 and CD56 in diagnostic immunohistochemical panels for SPNP.

Surgical resection is the mainstay of treatment even in the setting of metastatic or local spread. Two- and 5-year survival rates as high as 97% and 95%, respectively, have been reported.^8^ Invasion of either the portal vein or the superior mesenteric artery does not rule out surgical resection and there is a general understanding that surgical debulking may be warranted even in the context of metastatic disease due to a prolonged or indolent course. Around 10–15% of SPNPs develop metastasis and local recurrences have been rarely reported in the literature. Extensive efforts have been made to identify the pathological criteria predictive of metastatic or recurrent potential of a given SPNP tumor.

Tang et al.^9^ reported that solid/diffuse growth pattern with extensive tumor necrosis and high mitotic rate (>15 mitoses/50 high power field) were associated with an aggressive clinical course. Marchegiani et al.^1^ found expansive growth pattern, pancreatic parenchymal invasion, and capsular invasion to have a statistically significant association with recurrence of SPNP, whereas angiovascular or perineural invasion, nodal/liver metastasis, and margin status did not. Only 2 out of 131 patients included had a recurrence.^1^ One had a local recurrence in the pancreatic remnant with peritoneal metastasis 72 months after the initial pancreaticoduodenectomy, whereas the other patient developed liver and peritoneal metastasis 56 months after the initial distal pancreatectomy with splenectomy. Histological analysis showed pancreatic parenchymal invasion in both.

Strikingly, Memorial Sloan Kettering Cancer Center (MSKCC) reported a total absence of recurrence after an R0 resection in node-negative tumors.^10^ Local, vascular, or perineural invasion was not reported to be predictive of recurrence or overall survival. Irtan et al. reviewed all pediatric SPNP cases in France for a 20-year period and found that the only significant risk factors for recurrence were younger age (<13.5 years) at diagnosis and positive surgical margins at the initial resection.^11^ Nishihara et al. compared 19 nonaggressive SPNs with 3 aggressive SPNs and proposed that venous invasion, nuclear grade, and prominent necrotic areas were predictive of a more aggressive phenotype.^12^ In their case series of 71 SPNs, Yang et al. showed that vascular invasion, extrapancreatic invasion, nodal metastasis, and Ki67 index >4% were predictors of recurrence.^13^

Despite the remarkably typical appearance without any areas of necrosis or hemorrhage, the tumor described in this report had a clinically aggressive course with extensive local invasion. No significant nuclear pleomorphism or foci of sarcomatoid change were observed. It had a more diffuse growth pattern, and the mitotic activity was 6/10 high power field (Fig. 1E). The only features somewhat portentous of an aggressive course were the presence of vascular invasion and a higher Ki67 (5–6%), which have previously been reported to be associated with recurrence/metastasis although inconsistently. The recurrent tumor had a similar histological phenotype with a slightly higher mitotic activity (8–10/HPF), which was still well below the activity reported to be associated with invasion or recurrence.

Since SPNPs have a tendency to displace the surrounding structures rather than invading them, recurrences are usually resectable. There is paucity of data on the role of chemotherapy or radiation in either neoadjuvant or adjuvant setting and the limited data available are predominantly anecdotal. Strauss et al. described regression of an SPNP invading superior mesenteric vein with neoadjuvant cis-platinum and 5-fluorouracil, which was substantial enough to facilitate a surgical resection.^14^ Similarly, Maffuz et al. utilized seven cycles of gemcitabine in conjunction with radiation to downsize a locally advanced SPNP of head of the pancreas with extension to mesocolon, porta hepatis, and gastrocolic ligament.^15^ Machado et al. treated local recurrence in a patient with chemotherapy and the patient was reported alive 39 months from the index surgery. In their experience with treating metastatic SPNPs, Czarnecka et al. observed a partial response with FolFox-4 for 17 months until drug toxicity forced a switch to FOLFIRI, which unfortunately failed to curtail disease progression.^16^ Radiation alone was successfully used by Fried et al. and Zauls et al. to manage an unresectable SPNP.^17,18^ The case series by Law et al. describes 35 patients with SPNPs who received adjuvant chemotherapy and/or radiotherapy. Among these 24 had long-term follow-up, of which, 6 died of their disease, whereas 18 were alive at a mean interval of 51.1 months.^2^

Other treatment modalities that have been implemented for treating recurrent, metastatic, or nonresectable disease with some reported degree of success include radiofrequency ablation, transcatheter arterial embolization, transcatheter arterial chemoembolization, selective internal radiotherapy, and hyperthermic intraperitoneal chemotherapy.^19–22^ These reports notwithstanding, it bears reiteration that surgical resection is the definitive treatment. A better understanding of the factors associated with recurrence/metastasis would help determine the appropriate surveillance or adjuvant treatment if any are warranted.

## Abbreviations Used

CT: computed tomography
HPF: high power field
SPNP: solid pseudopapillary neoplasms of the pancreas
